# From gut dysbiosis to decidual hostility: the immuno-metabolic crosstalk driving recurrent pregnancy loss

**DOI:** 10.3389/fimmu.2025.1746620

**Published:** 2026-01-21

**Authors:** Yimin Shi, Xiufeng Tang

**Affiliations:** 1 Traditional Chinese Medicine Department, Shandong Provincial Maternal and Child Health Care Hospital, Jinan, China; 2 Department of Pharmacy and Shandong Provincial Key Traditional Chinese Medical Discipline of Clinical Chinese Pharmacy, Shandong Cancer Hospital and Institute, Shandong First Medical University and Shandong Academy of Medical Sciences, Jinan, China

**Keywords:** decidual NK cells, decidual microenvironment, gut dysbiosis, immunometabolism, metabolic reprogramming, recurrent pregnancy loss, regulatory T cells

## Abstract

Recurrent pregnancy loss (RPL), particularly its unexplained form (URPL), represents a formidable challenge in reproductive medicine. Although traditionally attributed to local immune imbalances at the maternal-fetal interface, this perspective may not fully account for the condition’s upstream etiological drivers and recurrent nature. This review transcends this limitation by proposing and systematically substantiating an integrative ‘gut-systemic-decidual’ model of immunometabolic dysregulation. We posit that a key pathological cascade in many URPL cases may originate with distal gut dysbiosis, which, through imbalanced metabolite profiles and the leakage of inflammatory molecules such as lipopolysaccharide (LPS), triggers systemic ‘metabolic endotoxemia’ and fundamentally reprograms the metabolic state of circulating immune cells. This systemic ‘first hit’ is compounded when these ‘pre-sensitized’ cells migrate to an equally metabolically stressed and ‘hostile’ decidual microenvironment—a ‘second hit’ characterized by hypoxia and high lactate. This culminates in the functional collapse of the core sentinels of maternal-fetal tolerance, namely regulatory T (Treg) and decidual natural killer (dNK) cells, due to profound metabolic misprogramming. Ultimately, this integrated model elevates the etiological understanding of URPL from a ‘local conflict’ to that of a ‘systemic disease,’ paving the way for the development of dynamic warning systems that integrate multi-omics data and for the design of multi-level precision intervention strategies targeting patient stratification and preventive approaches for the gut, systemic metabolism, and the local microenvironment.

## 1 Introduction

Recurrent pregnancy loss (RPL) is a major clinical challenge in reproductive medicine, inflicting not only physiological harm but also profound psychological trauma on patients. Despite decades of research, up to 50% of cases remain idiopathic and are classified as unexplained RPL (URPL) (1). It is crucial to recognize that URPL is likely not a single disease entity but rather a common clinical endpoint for a heterogeneous group of underlying pathologies. The immuno-metabolic dysregulation model proposed here may represent a significant, yet not exclusive, pathway contributing to a substantial subset of these cases. Among the many potential etiologies, immune dysregulation is widely considered a key factor strongly associated with URPL. The rationale for this hypothesis lies in the exquisite immunological paradox of a successful pregnancy: the maternal immune system must establish and maintain tolerance to the semi-allogeneic fetus, which carries paternal antigens, while simultaneously preserving its capacity to defend against pathogens (2, 3).

Traditionally, immunological research into RPL has focused on local imbalances in the proportions or functions of immune cells at the maternal-fetal interface, such as the Treg/Th17 cell axis (4). However, these studies often treat the uterus as an isolated local phenomenon, an approach that largely fails to explain the upstream drivers of this local immune dysfunction. Why does the program for establishing immune tolerance repeatedly fail in certain women? What are the root pathophysiological mechanisms?

In recent years, groundbreaking advances in the field of immunometabolism have offered a novel perspective. This field has revealed that the functional state of an immune cell is intricately linked to its intrinsic metabolic programming (5, 6). For instance, tolerogenic regulatory T (Treg) cells rely on oxidative phosphorylation (OXPHOS) to sustain their suppressive function, whereas pro-inflammatory T helper 17 (Th17) cells depend on aerobic glycolysis to support their rapid proliferation and effector functions (7, 8). This discovery implies that the immune cell imbalances observed in RPL may, at their core, represent a profound dysregulation of metabolic programming.

Building on this premise, this review aims to move beyond the confines of traditional local-centric immune studies by proposing an integrative ‘gut-systemic-decidual’ model of immunometabolic dysregulation. We hypothesize that URPL is not a problem confined to the endometrium but rather a systemic disease that originates with a distal homeostatic imbalance (e.g., in the gut), progresses through systemic immunometabolic reprogramming, and ultimately manifests at the maternal-fetal interface. This article will systematically elucidate how gut dysbiosis, as the initial ‘distal disturbance’, triggers a cascade that reshapes the maternal systemic immunometabolic landscape; how these functionally pre-conditioned immune cells, upon migrating into the decidua, encounter a ‘hostile microenvironment’ that is itself metabolically compromised; and how this dual systemic and local pressure ultimately drives the functional failure of key immune cells (notably Treg and dNK cells), thereby dismantling maternal-fetal immune tolerance. By integrating multi-dimensional, recent evidence, we aim to provide a more complete and in-depth explanation for the pathophysiology of URPL and to illuminate new directions for the development of novel diagnostic markers and precision preventive and therapeutic strategies.

## 2 Gut dysbiosis: the initiating factor of systemic immuno-metabolic dysregulation

A growing body of evidence suggests that the immune dysregulation in RPL is not a localized uterine event but that its pathophysiological roots can be traced to distant organ systems. a concept well-established in the context of the gut-skin and gut-brain axes (9, 10)**. Among these, the gut, as the body’s largest immune organ and microbial reservoir, is emerging as a focal point of investigation. A healthy gut microbiota and its metabolites, such as short-chain fatty acids (SCFAs), are critical regulators for maintaining systemic immune homeostasis (11). SCFAs not only provide energy to colonocytes and maintain intestinal barrier integrity but also directly regulate the differentiation and function of distant immune cells, such as T cells. Classic animal model studies have long established that SCFAs like butyrate, produced by commensal bacteria, are key factors in inducing the differentiation of colonic Treg cells (12, 13), thereby establishing a systemic environment of immune tolerance. A recent landmark study provided direct and compelling evidence for this “gut-immune” axis: in a clinical cohort of patients with unexplained RPL (URPL), gut dysbiosis and the associated reduction in microbial metabolites (such as SCFAs and secondary bile acids) were directly and significantly correlated with a reduction in protective peripheral Treg cells and an increase in pro-inflammatory Th1/Th17 cells (14). Deeper mechanistic investigations further revealed that SCFAs (particularly propionate and butyrate) can directly act on G protein-coupled receptors (GPR43) on the surface of T cells in distant immune organs (such as the spleen), potently promoting their differentiation into immunosuppressive Treg cells and consequently ameliorating a mouse model of fetal loss (15). This provides a clear mechanistic link for the concept that a healthy gut ecosystem actively cultivates a systemic immune tolerance environment favorable for pregnancy through its metabolic output.

However, when the gut microbiota homeostasis is disrupted (gut dysbiosis), this delicate balance collapses. The overgrowth of pathogenic bacteria and the decline of beneficial bacteria lead to a decrease in the production of beneficial metabolites like SCFAs, coupled with an increase in inflammatory molecules such as lipopolysaccharide (LPS) (4). This concept is supported by early evidence: two decades ago, prospective studies began to explore the association between host genetic susceptibility to LPS (such as CD14 gene polymorphisms) and the risk of RPL, laying the early groundwork for an “endotoxin hypothesis” (16). More importantly, dysbiosis is often accompanied by impaired intestinal barrier function, a condition clinically referred to as “leaky gut” (17, 18). This allows microbial products like LPS, which are normally confined to the intestinal lumen, to cross the barrier and enter the bloodstream, triggering a sustained, low-grade systemic inflammatory response (1, 18, 19) that defines “metabolic endotoxemia”. Notably, this pathological cascade may be more complex; for instance, some evidence suggests the existence of a “gut-lung-uterus” axis, where inflammatory signals originating from the gut may first affect the lungs and then extend to the uterus via the circulatory system, forming a multi-organ cascade (20). Furthermore, this gut-decidual axis may not be a one-way street. The profound hormonal shifts during pregnancy, including rising levels of progesterone and estrogen, can themselves modulate the composition and function of the gut microbiota. This suggests a potential bidirectional feedback loop, where pregnancy-induced changes in the gut could either reinforce a healthy state or, in susceptible individuals, exacerbate pre-existing dysbiosis, further amplifying the systemic inflammatory pressure on the maternal-fetal interface.

**Figure 1:**
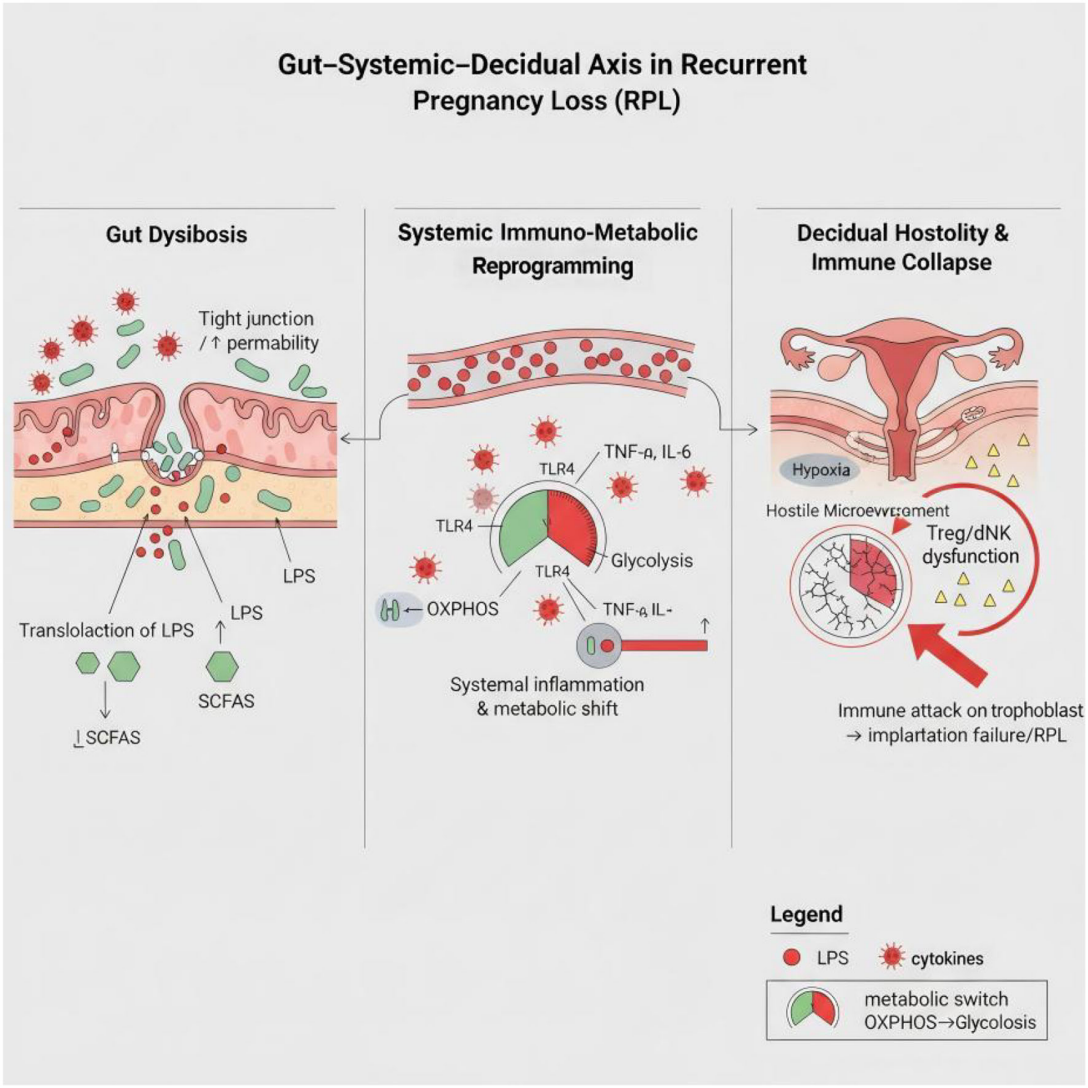
The ‘Gut-Systemic-Decidual’ immunometabolic axis model driving unexplained recurrent pregnancy loss (URPL). This model illustrates the proposed multi-stage pathophysiological cascade in URPL. (1) Gut Dysbiosis: An imbalanced gut microbiota leads to reduced production of beneficial short-chain fatty acids (SCFAs) and increased intestinal permeability (“leaky gut”). (2) Systemic Inflammation: This allows microbial products like lipopolysaccharide (LPS) to enter the circulation, triggering systemic low-grade inflammation and metabolically reprogramming circulating immune cells (e.g., T cells, NK cells) towards a pro-inflammatory state. (3) Decidual Hostility: These pre-sensitized immune cells migrate to the decidua, where a locally hostile metabolic environment (e.g., hypoxia, high lactate) further compromises their function, leading to the collapse of maternal-fetal tolerance and pregnancy failure.

Circulating LPS, as a potent pathogen-associated molecular pattern (PAMP), exerts widespread “pre-sensitizing” and “metabolic reprogramming” effects on the maternal immune system. The detrimental impact of this systemic inflammatory state is multi-faceted. First, in addition to inducing the production of large quantities of pro-inflammatory cytokines (such as TNF-α and IL-6) from monocytes/macrophages via the classic Toll-like receptor 4 (TLR4) signaling pathway (17), LPS can also activate the complement system. This leads to the generation of the potent inflammatory mediator C5a, which directly mediates placental injury and pregnancy loss through its receptor, C5aR1 (21). This chronic, low-grade inflammatory environment constitutes a “pre-sensitization” process for the maternal immune system, systematically altering the differentiation potential of T cells by inhibiting Treg cell differentiation while promoting the expansion of Th17 cells, thereby disrupting systemic immune balance (22). Second, at a deeper level, LPS fundamentally re-wires the metabolic programs of immune cells. Persistent LPS stimulation induces a shift toward glycolytic metabolism in myeloid cells like monocytes, a pro-inflammatory “war-ready” phenotype that renders them more easily activated to release inflammatory mediators (23). Within the cell, continuous LPS stimulation inhibits the activity of core metabolic sensors like AMPK and SIRT1. This not only drives cells toward a pro-inflammatory phenotype dependent on glycolysis but can also directly trigger a form of metabolism-dependent cell death termed “ferroptosis,” causing direct damage to tissues such as the endometrium (24). Furthermore, LPS can amplify inflammation at a post-transcriptional level through a newly discovered and elegant mechanism. It promotes the sequestration of RC3H1, a protein responsible for degrading TNF-α mRNA, within intracellular stress granules by a protein named MNSFβ. This sequestration prevents the degradation of TNF-α mRNA, leading to its uncontrolled expression and creating a self-amplifying inflammatory loop (25) (**Figure 1**).

## 3 The decidual microenvironment: from immune sanctuary to metabolic hostility

**Figure 2:**
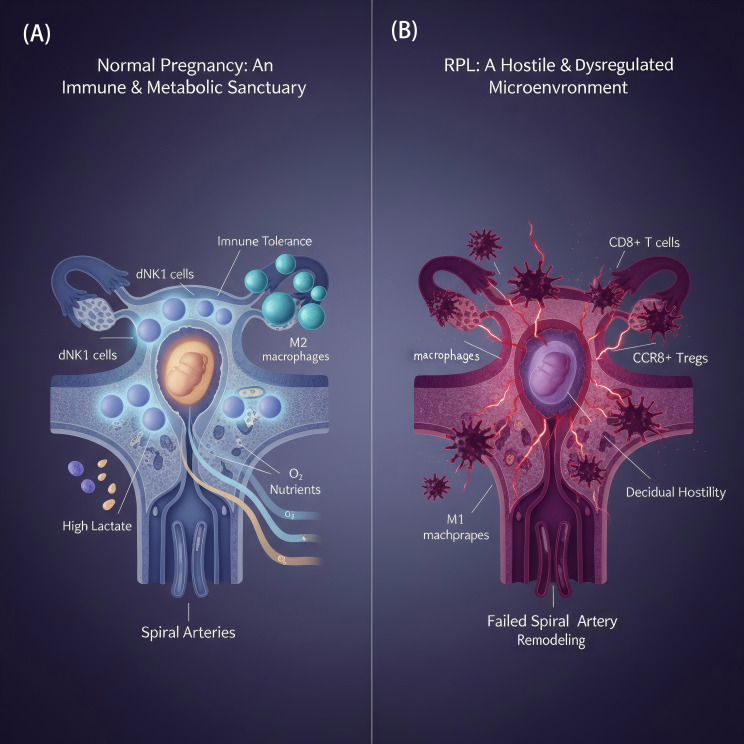
Schematic comparison of the decidual microenvironment in normal pregnancy and RPL. **(A)** Normal Pregnancy: A tolerogenic environment is actively maintained, characterized by an abundance of immunomodulatory cells such as decidual NK (dNK) cells, M2-like macrophages, and regulatory T (Treg) cells. Successful spiral artery remodeling ensures adequate oxygen and nutrient supply, supporting metabolic homeostasis. **(B)** Recurrent Pregnancy Loss (RPL): The microenvironment shifts towards hostility, with increased infiltration of cytotoxic CD8+ T cells and pro-inflammatory M1 macrophages. Defective vascular remodeling results in local hypoxia and accumulation of acidic metabolites like lactate, creating conditions unfavorable for embryonic development.

If gut dysbiosis is the “distant storm” that initiates the pathological process, then the decidual microenvironment is the local battlefield where this conflict ultimately culminates. Under physiological conditions, the early-pregnancy decidua is a unique immune-privileged microenvironment that actively suppresses immune attacks to protect the embryo through multiple mechanisms. Among these, decidual stromal cells (DSCs) play the role of central coordinators. They not only possess intrinsic immunosuppressive functions but also secrete various cytokines and chemokines to recruit and regulate the phenotype and function of immune cells (especially Treg cells and specialized dNK cells), collectively building a robust niche of immune tolerance (25) (**Figure 2**). This microenvironment is also metabolically unique: it is a relatively hypoxic yet nutrient-rich area, a distinctive metabolic feature crucial for maintaining immune tolerance (26, 27). Importantly, this privileged status is not a passive “isolation” but a dynamic equilibrium actively constructed and maintained by a sophisticated signaling network between maternal and fetal cells. For example, normal dNK cells secrete pigment epithelium-derived factor (PEDF) to protect DSCs from inflammatory and apoptotic damage, forming a positive protective feedback loop (28).

In patients with RPL, however, this “immune sanctuary” transforms into a metabolically hostile environment (29). This transformation is particularly evident in its spatial dimension. The latest spatial transcriptomics studies have clearly depicted that the implantation site (IZ) of a normal decidua is a protective zone enriched with dNK1 cells and M2-like macrophages. In RPL, however, this protective structure is dismantled; the protective cells are sharply reduced and replaced by an infiltration of cytotoxic CD8+ T cells. In essence, the spatial distribution pattern of cells undergoes a fundamental reversal (30). The root of this spatial collapse lies in the profound dysregulation of molecular and metabolic programs within the microenvironment. Multiple integrative transcriptomic and metabolomic studies have consistently revealed significant metabolic disturbances in the decidual tissue of RPL patients (14, 31, 32). Even at the upstream epigenetic level, RPL-associated villi and decidua exhibit a state of widespread DNA hypermethylation, affecting the gene expression of numerous key signaling pathways (33). This metabolic dysregulation is not merely an intrinsic cellular malfunction but is also driven by aberrant signaling molecules in the microenvironment. For instance, DSCs from RPL patients excessively secrete the extracellular matrix protein Decorin, which acts as a detrimental signaling molecule that directly targets decidual macrophages, inducing their mitochondrial dysfunction (34). Concurrently, systemic lipid imbalance further exacerbates the local pathology. It leads to a sharp increase in downstream inflammatory metabolites of arachidonic acid (AA) in the decidua—oxylipins such as PGE2 and PGF2α—and these inflammatory lipid molecules further shape a pro-inflammatory microenvironment (35).

Amid these chaotic metabolic changes, a hallmark feature is an aberrant upregulation of the glycolytic pathway and an impaired tricarboxylic acid (TCA) cycle, leading to the excessive accumulation of acidic metabolites like lactate (31, 32). It is noteworthy, however, that the dysregulation of glucose metabolism is not a simple “upregulation” or “downregulation” but rather a failure in the control of key metabolic nodes. A pioneering study discovered that the glycolytic intermediate fructose-1,6-bisphosphate (FBP) is a critical signal for inducing tolerogenic interactions between DSCs and macrophages, and its deficiency in RPL may be a significant cause of immune dysregulation (36). Furthermore, autophagy, a core mechanism for maintaining cellular metabolic homeostasis, is severely defective, preventing the timely clearance of damaged organelles and proteins and thus exacerbating cellular stress and dysfunction (37). This dysregulation can even trigger “ferroptosis,” a form of metabolism-dependent cell death, inflicting direct physical damage on decidual and trophoblast cells (23).

This metabolically disordered microenvironment not only directly harms maternal immune cells but also poses a grave threat to the survival and function of trophoblast cells, which are semi-allogeneic. At the same time, the disturbed microenvironment disrupts the intricate metabolic programs of the trophoblasts themselves. For example, the expression of HSD3B1, a key enzyme for progesterone synthesis in invasive extravillous trophoblasts (EVTs), is downregulated, leading to impaired local synthesis of progesterone, an endogenous immunosuppressant (38). The secretion of the critical growth factor GDF15 by trophoblasts may also be reduced, further weakening their invasive capacity (39). Even at a more refined molecular level, trophoblast function is severely disrupted by epigenetic and post-transcriptional dysregulation. For example, the abnormal upregulation of long non-coding RNAs (such as Lnc-HZ05) disrupts cytoskeletal rearrangement, thereby inhibiting the formation of migrasomes, a key structure for trophoblast migration (40).

More importantly, this hostile battlefield is not silent but is filled with erroneous signals and disrupted interactions. This “acidified” and “dysregulated” metabolic microenvironment, shaped by a multitude of factors (41), ultimately exerts a direct and decisive influence on the function of immune cells.

For example, high concentrations of lactate and a hypoxic environment can stabilize the expression of HIF-1α, which plays a dual role in different cells: on one hand, it inhibits the proliferation and function of Treg cells (42); on the other, it promotes the survival and differentiation of pro-inflammatory Th17 cells, thus directly driving the Treg/Th17 imbalance from a metabolic level. Meanwhile, other dysregulated metabolite profiles (such as abnormal tryptophan metabolism) can directly impair the cytotoxic regulation and vascular remodeling capabilities of dNK cells via pathways like the aryl hydrocarbon receptor (AHR) pathway (5). Single-cell sequencing has revealed with unprecedented precision that the decidua in RPL does not suffer from a uniform dysfunction but is instead dominated by pathogenic cell subpopulations. For instance, the massive emergence of “inflammatory” iDSCs and “glycolytic” glyDSCs actively secrete inflammatory factors, remodeling the entire communication network of the microenvironment (43). This breakdown in communication is also reflected between mother and fetus. Normally, fetal villi deliver an inhibitory signal, miR-29a-3p, to maternal dNK cells via exosomes to suppress their aggression. In RPL, however, this critical tolerance signal is significantly weakened, leading to a communication breakdown and the eruption of conflict (44). Therefore, when immune cells that have already been “pre-sensitized” in the systemic circulation arrive at a decidua filled with erroneous cell subpopulations, incorrect signaling molecules, and aberrant metabolic products, not only do they fail to be reprogrammed into a tolerogenic phenotype, but their inherent inflammatory propensity is further amplified and solidified, ultimately leading to an immune attack on the embryo.

## 4 Functional collapse of key immune cells: the fatal error in metabolic programming

If the deterioration of the decidual microenvironment sets the local stage for the conflict, then the functional collapse of key immune cells within this environment is the direct cause of pregnancy failure. Here, we will focus on two core cell types essential for maintaining maternal-fetal tolerance—regulatory T cells (Tregs) and decidual natural killer (dNK) cells—to dissect how profound errors in their metabolic programming lead to their functional collapse in RPL.

### 4.1 Insufficiency of regulatory T cells

Treg cells are the central regulators of maternal-fetal immune tolerance (45). In RPL, their functional deficiency is not merely a correlative phenomenon but has a clear causal relationship. Large-scale Mendelian randomization studies have confirmed that a reduction in specific Treg subsets (such as CD39+ resting Tregs) is a root cause of RPL (46). This insufficiency manifests as a dual decline in both quantity and quality, and may ultimately lead to a defect related to immune memory.

**Figure 3:**
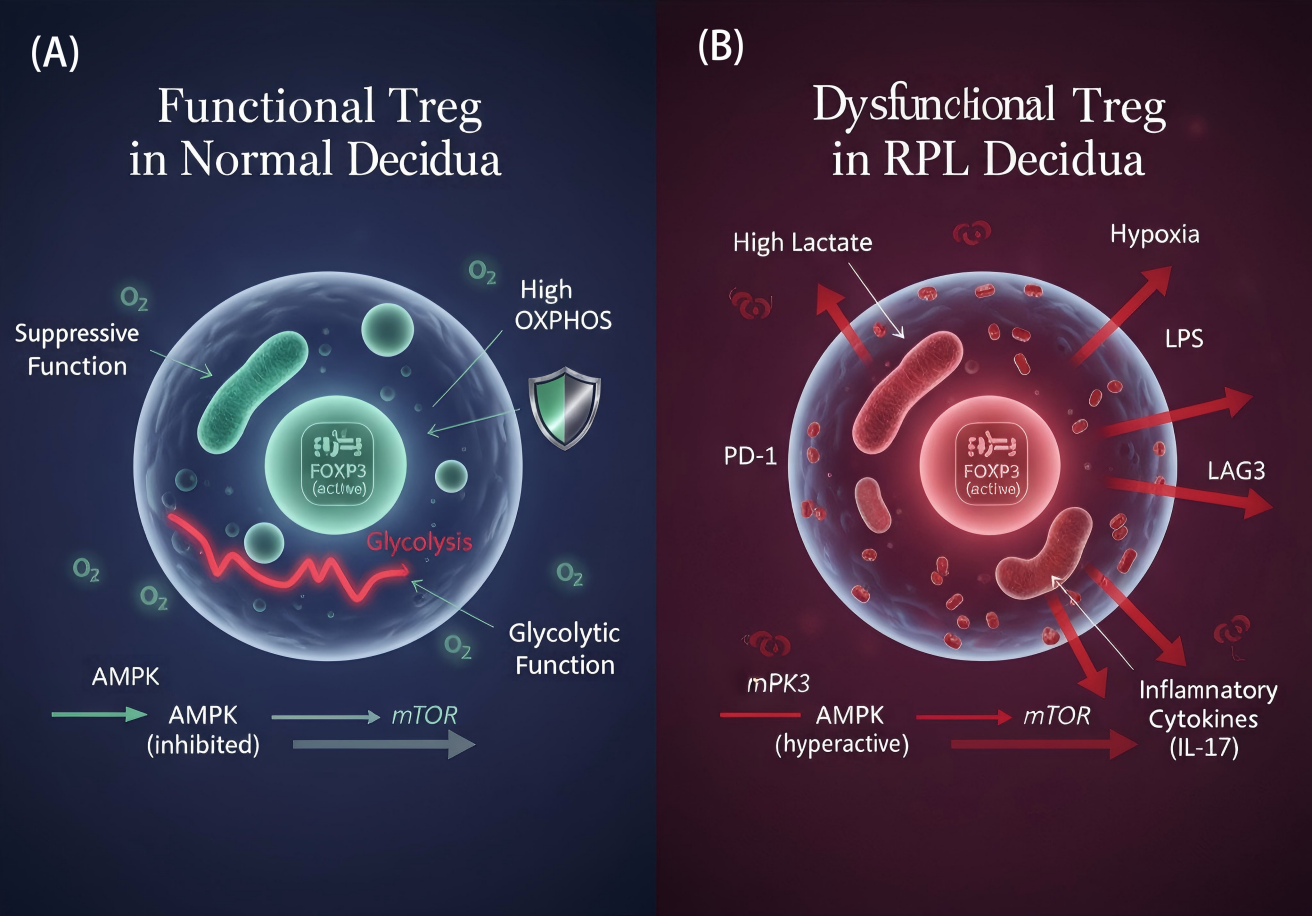
Function and metabolic programming of Treg cells in the normal and RPL decidual microenvironment. **(A)** Functional Treg in Normal Pregnancy: Treg cells rely on mitochondrial oxidative phosphorylation (OXPHOS) for sustained FOXP3 expression and immunosuppressive function, a state supported by active AMPK and suppressed mTOR signaling. **(B)** Dysfunctional Treg in RPL: In the hostile RPL microenvironment (high lactate, hypoxia, LPS), Treg metabolism is rewired towards glycolysis. This shift, driven by suppressed AMPK and activated mTOR signaling, leads to unstable FOXP3 expression, upregulation of exhaustion markers (e.g., PD-1), and impaired suppressive capacity, with a potential to convert into IL-17-producing pathogenic cells.

In terms of quantity, while the conventional view holds that the overall proportion of Treg cells is decreased in RPL (4), high-resolution mapping with single-cell technologies reveals that the loss is not of generic Tregs, but of specific subsets that exert critical effector functions. One study identified a CCR8+ Treg subset, specifically enriched in the decidua, as the core force maintaining local immunosuppression. This subset is significantly reduced in RPL, and its adoptive transfer in a model system can effectively rescue pregnancy, directly demonstrating its causal role. This loss can be traced to upstream signaling defects, such as reduced expression of the key recruiting chemokine CCL1 (47), or the inability to suppress miR-520a-5p due to the downregulation of circular RNA circDDX21, leading to the abrogation of expression of the master Treg transcription factor FOXP3 at its source (48). In terms of quality, even the Tregs that remain often fall into a state of functional exhaustion. Similar to the tumor microenvironment, Treg cells in the decidua of RPL patients exhibit high expression of a series of inhibitory receptors, including PD-1 and LAG3, signifying a loss of their sustained suppressive capacity (49) (**Figure 3**).

Underlying this functional collapse is a profound error in metabolic programming. The suppressive function of Tregs is highly dependent on the sustained energy provided by mitochondrial oxidative phosphorylation (OXPHOS) and the metabolic adaptability mediated by FOXO1 (45). However, in the “acidified” and “hypoxic” decidual microenvironment of RPL, the inherent “glycolytic fragility” of Treg cells makes it difficult for them to adapt (50, 51). This metabolic reprogramming is actively driven by dysregulated signaling pathways: the energy sensor AMPK pathway is suppressed, while the mTOR pathway, which promotes glycolysis, is abnormally activated. This imbalance not only inhibits Treg differentiation but also simultaneously promotes the generation of pro-inflammatory Th17 cells, and may even induce the conversion of Tregs into pathogenic, IL-17-secreting cells (52). At the organellar level, endoplasmic reticulum (ER) stress, a core hallmark of metabolic homeostasis imbalance, is also closely associated with Treg functional defects, forming a critical link between metabolic pressure and the collapse of Treg function (53).

However, the reduction in quantity and decline in quality still cannot fully explain the “recurrent” nature of RPL. Recent discoveries regarding Treg immune memory offer a possible explanation. Under normal circumstances, maternal tolerance to the fetus is a memory-like response, mediated by cells known as “ex-Tregs”—cells that were once Tregs but lost FOXP3 expression postpartum. In a subsequent pregnancy, these ex-Tregs can be rapidly reactivated to re-express FOXP3 and re-establish immune tolerance. This reveals that the plasticity of FOXP3 expression is key to successful pregnancy-related immune memory (54). Based on this, a hypothesis can be proposed: the root cause of the repeated failures in RPL may lie in a defect of this “plasticity” and the exhaustion of the “memory cell pool.” Fetal Tregs themselves exist in an unstable, “hypofunctional” state (51). It is conceivable that in the pathological microenvironment of RPL, this instability is amplified, causing Tregs to easily lose FOXP3 expression and undergo premature apoptosis or conversion. More critically, persistent metabolic stress may lead to the epigenetic state of these cells becoming fixed, rendering them unable to be effectively reactivated in subsequent pregnancies, thereby causing a loss of “tolerance memory.” This theory systematically integrates the phenomena of “reduced quantity,” “functional exhaustion,” and “metabolic instability,” providing a new theoretical framework to explain the recurrent nature of RPL.

### 4.2 Dysfunction of decidual natural killer cells

dNK cells are the most abundant immune cells in the early pregnancy decidua, and their functional prominence in both healthy pregnancy and infertility is well-documented (55). Their core task is to transition from a cytotoxic phenotype in the peripheral blood to a functional phenotype that promotes trophoblast invasion and spiral artery remodeling (6, 55). In RPL, however, this identity remodeling fails, and their function reverses from promoting angiogenesis and immune tolerance to exerting cytotoxic and pro-inflammatory effects.

First, this functional reversal is clearly visible in the macroscopic landscape of cell subsets. Multiple single-cell sequencing studies have consistently shown that in the decidua of RPL patients, the population of subsets with immunomodulatory and pro-angiogenic functions, represented by CD39+ dNK1 cells, is sharply reduced. In contrast, dNK3 and CD18+ dNK subsets, which have pro-inflammatory and cytotoxic potential, undergo abnormal expansion (56, 57). The root of this dramatic shift is a combination of intrinsic cellular factors and the external environment. The intrinsic factor is an imbalance in the transcriptional program of dNK cells, where transcription factors driving tolerance (such as RELB) are suppressed, while those driving inflammation, such as T-bet (TBX21), are activated (58). The external factor is induction by the pathological microenvironment; as previously mentioned, inflammatory stromal cells (iDSCs) directly induce the emergence of pathogenic dNK subsets through spatial interactions (43).

At the heart of this collapse is, again, a profound error in metabolic programming. Normal dNK cells are highly dependent on OXPHOS to maintain their pro-angiogenic and immunomodulatory functions, and their metabolic state is strictly regulated by mTORC1 signaling. In RPL, however, mTORC1 signaling activity is significantly suppressed, leading directly to the collapse of the dNK cell metabolic program (59). The core of this metabolic dysfunction points directly to the mitochondria—the latest evidence has directly linked dNK cell dysfunction to the downregulation of GRIM19, a key protein in the mitochondrial respiratory chain, which constitutes direct evidence of damage to their functional foundation (60).

Ultimately, this comprehensive collapse, spanning from upstream transcription to core metabolism, leads to a twofold deterioration of dNK cell function:

Complete loss of pro-angiogenic and immunomodulatory functions: The loss of the critical CD39+ dNK subset (which supports trophoblasts by secreting M-CSF) has been confirmed to be causal; its functional defect can lead to pregnancy loss in humanized mouse models, which can be rescued by the adoptive transfer of this subset (61). Concurrently, inhibitory signals from trophoblasts, such as exosomal miR-185-5p, can directly inhibit the secretion of vascular endothelial growth factor (VEGF) by dNK cells, thereby blocking spiral artery remodeling (62).

Abnormal activation of cytotoxic and pro-inflammatory functions: The cytotoxicity of dNK cells is activated under the stimulation of an abnormal metabolic microenvironment (e.g., imbalanced tryptophan metabolites) and inflammatory signals (5). One theoretical model suggests that metabolic stress (such as ER stress) can downregulate inhibitory ligands (HLA-C/G) on the surface of trophoblasts, allowing dNK cells to recognize and attack them (63). The elevated levels of Granzyme B in the decidua are direct evidence of this phenomenon (32). At the same time, dNK cells switch to secreting large amounts of pro-inflammatory cytokines such as IFN-γ. This loss of control stems from the failure of multiple inhibitory mechanisms: at the intercellular level, inhibitory exosomal signals from the fetus (such as miR-29a-3p) are interrupted (64); at the intracellular level, the expression of the inhibitory miR-122-5p is downregulated, rendering it unable to suppress the pro-inflammatory transcription factor T-bet (65); and alterations in their surface activating receptor profile (such as NKp46) further exacerbate the pro-inflammatory shift (66).

In summary, the functional collapse of dNK cells is a comprehensive process of dysregulation—from transcription and metabolism to subset structure—driven by both distant and local factors. This functional reversal is a key execution step leading to insufficient embryonic blood supply and direct immune rejection.

## 5 Diagnostic and therapeutic perspectives: from multidimensional warning to precision intervention

### 5.1 Building a multidimensional warning system: integration of immuno-metabolic targets

The diagnosis of RPL is gradually evolving from a traditional model reliant on single, static indicators like the Treg/Th17 ratio, whose limited predictive power fails to capture the full picture of the disease (4, 17, 19). As noted in commentaries on the field, the key to building robust predictive models for RPL lies in integrating multi-omics data and deeply exploring the metabolic reprogramming of immune cells (67). Based on this, a new paradigm for a multidimensional warning network that integrates immune status, metabolic fingerprints, and cellular function is emerging.

This paradigm shift has rapidly moved from theory to practice. A predictive model that integrates routine peripheral blood immune and metabolic indicators using machine learning can now predict subsequent pregnancy outcomes with high accuracy (AUC = 0.88), marking a significant advance in macro-level prediction (68). In the realm of non-invasive diagnostics, the use of urine metabolic fingerprints combined with machine learning has enabled rapid, high-throughput screening for RPL, with a diagnostic efficacy (AUC = 0.91) that offers a feasible path for large-scale early warning (69). Beyond mere prediction, this new approach can also achieve deeper mechanistic subtyping. Studies have confirmed a direct link between amino acid metabolism disorders in the serum of RPL patients and Th1/Th2 immune imbalance, and a diagnostic model built on this association has demonstrated extremely high accuracy (AUC = 0.969) (70).

More precise diagnosis requires delving into the key site of the pathological process—the maternal-fetal interface. With unprecedented resolution, spatial transcriptomics has revealed a reduction in protective cell subsets and an infiltration of aggressive cells in the RPL decidua, identifying FOSL2 as a core transcription factor driving this pathological shift and positioning it as a highly promising diagnostic target (30). At the cellular level, the functional defects of key immune cells are being pinpointed to specific molecular events. For instance, the dysfunction of dNK cells has been shown to be closely related to the downregulation of the mitochondrial protein GRIM19, suggesting that GRIM19 could serve as a cellular-level indicator for assessing dNK cell function (60). Similarly, in-depth studies of Treg cell metabolism have revealed the central role of the energy sensor LKB1 and the mitochondrial uncoupling protein UCP3 in maintaining their function, providing a theoretical basis for developing mechanism-based diagnostics based on Treg metabolic status (71, 72). These mechanistic discoveries also offer new tools for evaluating existing therapies; for example, monitoring changes in the metabolic profiles of patients before and after lymphocyte immunotherapy (LIT) may emerge as a novel diagnostic strategy for dynamically assessing therapeutic efficacy and prognosis (73).

A truly comprehensive diagnostic model must expand its perspective to include paternal contributions. The latest research has extended the etiology of RPL to the level of paternal epigenetics, discovering that abnormal m6A modification of sperm mRNA is associated with early embryonic arrest, thus providing a completely new molecular target for diagnosis (74). From a metabolic standpoint, a taurine metabolism defect has also been identified in the sperm of male partners of RPL patients, which is not only a new biomarker but also hints at potential intervention strategies (75).

In summary, the future of RPL diagnosis is evolving from a linear assessment of single indicators to a multi-level, dynamic warning network. This network aims to integrate a portfolio of biomarkers spanning immunology, metabolomics, genetics, and epigenetics, thereby enabling a paradigm shift from “unexplained” to “precision diagnosis.” Several recent reviews have systematically organized this emerging biomarker system, providing a solid theoretical foundation for constructing the next generation of predictive frameworks (76, 77).

### 5.2 Precision intervention: new therapeutic strategies targeting immuno-metabolism

The new understanding of RPL pathophysiology based on the “gut-systemic-decidual” immuno-metabolic axis is giving rise to a series of novel therapeutic strategies, moving beyond traditional approaches to explore targeted immunomodulation for alloimmune-related RPL (78, 79). These strategies aim for multi-level, precision intervention, from the source and system to the local environment. Reviews have clearly pointed out that targeting microbiota-mediated immune regulation can open new avenues for RPL treatment (80).

#### 5.2.1 Reshaping the source: gut microbiome intervention

The first level of intervention directly targets the initiating step of the pathological process—the gut. The core principle is to restore the production of key metabolites (especially SCFAs) by remodeling the gut microbiota, thereby regulating the systemic immune response. Basic research has confirmed that the maternal gut microbiota and its product, SCFAs, are prerequisites for normal placental development (81). In abortion-prone models, direct supplementation with butyrate not only restores Treg cell numbers but also effectively reduces the rate of pregnancy loss, directly validating the efficacy of “replenishing key metabolites” (82). Clinically, high-quality randomized controlled trials (RCTs) have demonstrated that prebiotics can effectively increase beneficial bacteria and SCFAs in the gut of both mothers and infants (83). While probiotics and fecal microbiota transplantation are promising approaches, their application requires a cautious, strain-specific, and individualized strategy to avoid potential risks (84).

#### 5.2.2 Recalibrating the system: systemic immuno-metabolic therapies

The second level of intervention directly targets the dysregulated systemic immuno-metabolic network, aiming to correct the metabolic programs of immune cells and optimize the uterine microenvironment. The core of this strategy is to target key regulatory nodes of cellular metabolism. Clinical evidence shows that correcting the Th17/Treg imbalance in RIF patients with the mTOR inhibitor rapamycin (sirolimus) can significantly increase the live birth rate, providing direct evidence for “targeting metabolic pathways to correct immune imbalance” (85). Another key metabolic sensor, PPARγ, can be indirectly activated through dietary supplements (such as stigmasterol) to reprogram T cell metabolic patterns and restore the Treg/Th17 balance (86). Furthermore, the use of the classic drug metformin and novel SGLT2 inhibitors can ameliorate metabolic disorders in high-risk RPL populations by regulating pathways such as AMPK. At the same time, the newly discovered biomarker TRAF3, due to its key role in immuno-metabolism, offers new possibilities for assessing RPL risk and the potential efficacy of related therapies (87, 88).

In addition to regulating key nodes, directly supplementing critical metabolic substrates or activating endogenous defense systems is equally important. Supplementation with NAD^+^ precursors (NR or NMN) has been shown to restore NAD^+^ levels, improve mitochondrial function, and thereby rescue oocyte quality associated with maternal aging or metabolic diseases (89, 90). In tackling oxidative stress, the strategy has shifted toward activating the body’s own NRF2/HO-1 antioxidant system with micronutrients like N-acetylcysteine (NAC), a strategy proven to mitigate ROS-mediated fetal injury induced by environmental toxins (91, 92). Additionally, some pleiotropic regulators have shown potential. Melatonin has been shown to block the TLR4/MAPK inflammatory pathway in the placenta by remodeling the gut microbiota, reflecting an intervention strategy that targets the “gut-placenta axis” (93). Supplementing with L-arginine can optimize uterine-placental blood perfusion by increasing NO synthesis, creating a favorable physical environment for embryonic development (94).

#### 5.2.3 Reprogramming the endpoint: decidual local immunomodulation

The final level of intervention focuses on the maternal-fetal interface, aiming to directly reprogram dysfunctional immune cells or regulate their key signals. At the molecular level, activating the PD-1/PD-L1 immune checkpoint pathway can inhibit macrophage glycolysis and drive their differentiation toward a protective M2 phenotype, suggesting that PD-1 agonists may become a future therapy to mimic normal pregnancy signals and restore decidual immune homeostasis (95). At the cellular level, classic lymphocyte immunotherapy (LIT) has been shown in multiple clinical trials to effectively increase live birth rates by remodeling the systemic and local immune environment, including reducing the proportion of cytotoxic NK cells and reversing Th1/Th2 and Th17/Treg imbalances (73, 96).

Finally, the significance of all these intervention strategies extends beyond maintaining a single pregnancy. Critical evidence indicates that SCFAs produced by the maternal gut microbiota can cross the placenta and directly participate in the development and metabolic programming of the fetal cardiovascular, nervous, and immune systems. Therefore, targeting the regulation of maternal immuno-metabolic homeostasis during pregnancy is essentially a forward-looking safeguard for the long-term health of the offspring, carrying profound transgenerational significance (97).

## 6 Conclusion and future perspectives

This review has systematically challenged the traditional view of unexplained recurrent pregnancy loss (URPL) as an isolated uterine event. By integrating the latest evidence from immuno-metabolism, microbiology, and maternal-fetal medicine, we have constructed and substantiated a novel “gut-systemic-decidual” immuno-metabolic dysregulation model. We propose that URPL is not merely a local immune imbalance but a systemic disease initiated by distant gut dysbiosis, propagated through the metabolic reprogramming of systemic immune cells and inflammatory signaling, and ultimately erupting in a metabolically dysregulated decidual microenvironment. From the impaired systemic differentiation of Treg cells due to reduced gut SCFAs, to the “metabolic endotoxemia” triggered by LPS leakage; from the “metabolic hostility” of the decidual microenvironment, to the “functional collapse” of key immune cells (Tregs and dNK cells) due to errors in metabolic programming—this series of interconnected pathological events collectively dismantles maternal-fetal immune tolerance. This integrated model not only provides a deeper, more logical pathophysiological explanation for up to 50% of “unexplained” RPL cases but also opens up entirely new horizons for future research and clinical practice. It is important to acknowledge, however, the limitations of the current evidence. Much of the human data supporting this model is correlational, and future longitudinal studies and intervention trials are essential to firmly establish causality. Moreover, given the heterogeneity of URPL, this model likely explains a significant subset of cases, and the potential for bidirectional interactions within the gut-decidual axis warrants further investigation.

Looking ahead, based on this new paradigm, research in the RPL field will enter a new era of multidimensional, dynamic, and precision-based approaches.

In diagnostics, the future goal is to build a dynamic warning system capable of “full-temporal, full-spatial” risk assessment. This will require moving beyond the current static multi-omics snapshots to develop artificial intelligence (AI) predictive models that can integrate gut microbiota, serum metabolites, the epigenetic status of immune cells, and their real-time functions (such as Treg plasticity and memory status). This approach will be crucial for patient stratification, distinguishing subtypes of URPL based on their primary driver (e.g., ‘gut-driven inflammatory type’ vs. ‘local decidual metabolic type’). We envision that the management of high-risk pregnant women in the future may involve creating their personalized “Digital Twin” models, enabling pre-emptive warnings and guiding targeted preventive approaches through dynamic monitoring and algorithmic prediction before the cascade of immune imbalance is triggered. Furthermore, incorporating paternal factors (such as sperm epigenetic and metabolic defects) as standard parameters into these models will be a critical step toward achieving a truly comprehensive diagnosis.

In therapeutics, intervention strategies will shift from symptomatic treatment to multi-target, mechanism-based precision interventions. Future treatment regimens will be a multidimensional combination, potentially including: (1) “Engineered probiotics”: not just simple strain supplementation, but “Live Biotherapeutic Products (LBPs)” that are engineered to produce specific SCFAs or immunomodulatory molecules; (2) “Treg memory remodeling therapy”: using targeted drugs or cell therapies to repair the potentially depleted “ex-Treg” memory cell pool in RPL patients, restoring their rapid response capability in subsequent pregnancies; and (3) “Decidual targeted delivery systems”: using nanoparticles and other carriers to precisely deliver PD-1 agonists, metabolic regulators, or anti-inflammatory drugs to the maternal-fetal interface, allowing for highly selective intervention in the local microenvironment without affecting systemic immune function. These interventions represent promising preventive approaches that can be tailored to patient subgroups identified through advanced diagnostics.

In basic research, several core scientific questions remain to be answered. We need higher-resolution spatiotemporal multi-omics technologies to track the migration trajectory and metabolic state evolution of individual immune cells from the gut to the decidua in real time. We need more sophisticated humanized mouse models and decidual organoids to validate the causal relationships of key molecules in a setting that more closely resembles the human body. Most importantly, we need to answer an ultimate question: How do we define and maintain an immuno-metabolic homeostasis that is “most favorable for pregnancy”? This is not only for a successful pregnancy but also because the maternal immune-metabolic state during gestation profoundly influences the long-term health of the offspring through “metabolic programming”.

In conclusion, by expanding the etiological understanding of RPL from the local uterine environment to a systemic “gut-systemic-decidual” framework, we not only provide a critical new perspective for understanding this stubborn clinical challenge but also point toward a clearer research direction for safeguarding the long-term health of both mother and child.
